# Comparative transcriptome analysis of *Eogammarus possjeticus* at different hydrostatic pressure and temperature exposures

**DOI:** 10.1038/s41598-019-39716-y

**Published:** 2019-03-05

**Authors:** Jiawei Chen, Helu Liu, Shanya Cai, Haibin Zhang

**Affiliations:** 1 Institute of Deep-sea Science and Engineering, Chinese Academy of Sciences, Sanya, 572000 China; 2 University of Chinese Academy of Sciences, Beijing, 100049 China

## Abstract

Hydrostatic pressure is an important environmental factor affecting the vertical distribution of marine organisms. Laboratory-based studies have shown that many extant shallow-water marine benthic invertebrates can tolerate hydrostatic pressure outside their known natural distributions. However, only a few studies have focused on the molecular mechanisms of pressure acclimatisation. In the present work, we examined the pressure tolerance of the shallow-water amphipod *Eogammarus possjeticus* at various temperatures (5, 10, 15, and 20 °C) and hydrostatic pressures (0.1–30 MPa) for 16 h. Six of these experimental groups were used for transcriptome analysis. We found that 100% of *E. possjeticus* survived under 20 MPa at all temperature conditions for 16 h. Sequence assembly resulted in 138, 304 unigenes. Results of differential expression analysis revealed that 94 well-annotated genes were up-regulated under high pressure. All these findings indicated that the pressure tolerance of *E. possjeticus* was related to temperature. Several biological processes including energy metabolism, antioxidation, immunity, lipid metabolism, membrane-related process, genetic information processing, and DNA repair are probably involved in the acclimatisation in deep-sea environments.

## Introduction

Hydrostatic pressure is thought to be the major environmental factor that limits the vertical distribution of extent marine animals^1,2^. Studies in the western North Atlantic^3,4^ revealed a unimodal diversity-depth pattern. Diversity and biomass are relatively low at the upper bathyal and abyssal depths but peak at the intermediate depths, ranging from 1,000 m to 3,000 m^5^. This kind of depth zonation may have resulted from the colonisation of the deep sea by shallow-water organisms (for review see Brown & Thatje 2014^6^).

The colonisation of the deep sea occurred throughout selection and during the slow genetic drift of species that gradually adapted to life in the deep^6^. However, depth range shifts, which may be as important as geographic range shifts, are observed in response to ocean warming^7,8^. Thus, the ability of a shallow-water animal to acclimatise to deep-sea environments during its lifetime is vital. The combined effects of temperature, pressure, and oxygen concentration, may have constrained the bathymetric migrations of marine fauna^8^. Substantial studies have examined the tolerance of shallow-water invertebrates to high hydrostatic pressure and low temperature (for review see Brown & Thatje 2014^6^). These studies indicated that many extant marine benthic invertebrates can tolerate hydrostatic pressure outside their known natural distributions, and a low temperature could impede high pressure acclimatisation. A few studies have focused on gene expression, such as that of heat shock protein 70 (*hsp70*), in response to high pressure exposure^9–11^. However, the transcriptomic approach is seldom applied to relevant studies. A set of genes, which may be activated in response to high hydrostatic pressure (HHP) exposure, can be detected through comparative transcriptome analysis. Thus, the molecular mechanisms that enable shallow-water organisms to acclimatise to deep-sea environments can be studied.

Transcriptome analysis has been applied widely to discover mRNA-level responses, revealing the genetic basis of adaptation to deep-sea environments^12–15^. Most comparative transcriptome analyses of invertebrates were based on the comparisons of congeneric species that have different vertical distribution profiles, and common patterns of adaptation appeared in widely different taxa of deep-living organisms^16^. Many biological processes are possibly related to deep-sea adaptation, including alanine biosynthesis^15^, antioxidation^13,17^, energy metabolism^15,18^, immunity^18,19^, fatty acid metabolism^20^, and genetic information processing^15^. However, transcriptomic studies have seldom examined how shallow-water invertebrates acclimatise to a simulated immersion in the deep-sea. Applying transcriptome analysis to reveal deep-sea environmental acclimatisation is of great importance because both evolutionary adaptation and phenotypic acclimation are essential for high pressure adaptation^21^.

The impacts of hydrostatic pressure on shallow-living organisms were explored in many studies (for review see Somero 1992^22^). Lipid bilayer is regarded as one of the most sensitive molecular assemblages of hydrostatic pressure^21–25^. High pressure leads to a reduction of membrane fluidity; consequently, the physiological function of membrane, including potential transmission^23,26^, transmembrane transportation, and cell movement^27,28^, are impeded. The effects of HHP and low temperature are similar^29,30^. Parallel effects can be detected according to membrane composition with an increase in hydrostatic pressure of 100 MPa and a reduction in temperature of 13–21 °C^22^. Studies on microorganisms have indicated that the increasing proportion of unsaturated fatty acid and branched-chain fatty acid can remit the reduction of membrane fluidity imposed by high pressure and low temperature^27,31^. The structure of protein is depolymerised under high pressure, which results in the inactivation of enzymes^27,32,33^. Low temperature also has a negative effect on protein structure. Therefore, it induces elevated protein chaperoning^34,35^, which decreases the stabilisation of the secondary structures of RNA and DNA. Moreover, high pressure can strengthen hydrogen bonds. Consequently, processes that include DNA replication, transcription and translation, are impeded^36^.

The amphipod *Eogammarus possjeticus*, belonging to the gammaridean crustaceans, is widely distributed in the coastal and estuarine areas of the Yellow Sea and Bohai Sea in northern China^37^. The optimal temperature and salinity of *E. possjeticus* are 20–25 °C and 5–35, respectively^37^. Amphipods are not only ubiquitous in shallow water, but are also widespread at the hadal depth^38^, which suggests that shallow-water amphipods could acclimatise to HHP. In the present study, we examined the hydrostatic pressure tolerance of *E. possjeticus* for the first time. We employed comparative transcriptome analysis on *E. possjeticus* to examine their molecular responses to hydrostatic pressure stress at different temperature conditions. Our findings may shed light on the mechanisms behind molecular acclimatisation to HHP at the genetic level.

## Methods

### Collection, maintenance, and rearing of *E. possjeticus*

Adult specimens of *E. possjeticus* were net caught from an aquaculture farm in Shandong, China on December 2016. They were maintained at a closed recirculating aquacultural system (seawater was partially refreshed once a week) in the laboratory of the Institute of Deep-sea Science and Engineering, Chinese Academy of Sciences. The amphipods were reared in aerated filtered seawater (salinity: 34.7–35.3, 1 μm filtered, natural light cycle), and were fed with brine shrimp flakes thrice a week; unconsumed food was removed after 24 h. Experimental individuals were starved for 3 d prior to pressurisation.

The experimental animals were maintained in the aquacultural system at 20 °C for 2 weeks to acclimatise them to laboratory environments and allow them to recover from collection and handling stress. The other amphipods remained in the aquacultural system at a constant temperature, while 10 individuals were chosen from the aquacultural system and used for pressure incubation. After the 20 °C experiments were finished, which took at least a week, the aquacultural system temperature was adjusted to 15 °C by a maximum of 2 °C per day. All experimental samples acclimatised to the temperature after it reached 15 °C for at least 5 d before pressurisation. The same procedure was followed for the experiments conducted at 10 °C and 5 °C. Studies on temperature acclimation of insect^39–42^ and amphibians^43,44^ indicated that nearly all studied animals can acclimatise to a temperature variation of 5 °C within 5 d. Thus, the temperature acclimatisation of experimental samples in this study is likely to be reached before pressure incubation was achieved.

### Pressurisation

The hydrostatic pressure system was set to desired temperature by using circulating water bath (Tianheng Instrument Factory, Zhejiang, China) at least 6 h prior to each experiment. Ten adult individuals of similar size (length: 13 ± 2 mm) were placed inside the stainless pressure chamber (volume: ~20 litres, internal diameter 20 cm, internal depth 65 cm) which is full of filtered seawater at a constant temperature (±0.8 °C), and maintained for 1 h to allow acclimatisation and recovery from handling stress. Then, the pressure vessel was pressurised at 1 MPa per minute to experimental pressure by using hydraulic pump (AILIPU Science and Technology Co., Inc., Zhejiang, China). Experimental samples would be maintained at temperature (5, 10, 15, and 20 °C) and hydrostatic pressure (5, 10, 15, 20, 25, and 30 MPa) conditions for 16 h. The pressure chamber was sealed and isolated during this time period. The pressure was released instantaneously after hydrostatic pressure exposure for 16 h, and the samples were removed from the pressure chamber and snap frozen in liquid nitrogen. The maximum time elapsed between departure from experimental pressure and flash freezing is 3 min. The flash-frozen individuals were stored at −80 °C for further use. Muscle tissues were not dissected before RNA extraction.

Dissolved oxygen, salinity and pH value were measured by using YSI Professional Plus (YSI Inc., USA) to ascertain the stabilisation of seawater quality. In addition, concentration of nitrite nitrogen (NO_2_-N), ammoniacal nitrogen (NH_3_-N) and nitric nitrogen (NO_3_-N) were measured by using HACH DR 1900 (HACH Company, USA) before and after each experiment. No significant difference was found between the experimental context before pressurisation (dissolved oxygen: 5.38 ± 0.3 mg.L^−1^, salinity: 35.0 ± 0.3, pH: 8.1 ± 0.1, NO_2_-N: 0.0055 ± 0.0005, NH_3_-N: 0.015 ± 0.005, NO_3_-N: 0.015 ± 0.005) and after pressurisation (dissolved oxygen: 5.26 ± 0.4 mg.L^−1^, salinity: 35.0 ± 0.3, pH: 8.1 ± 0.1, NO_2_-N: 0.007 ± 0.0005, NH_3_-N: 0.02 ± 0.005, NO_3_-N: 0.015 ± 0.005).

### RNA extraction, quantification, and qualification

Approximately 50 mg pooled muscle tissues from 5 individuals of the same experimental group were used for each RNA extraction. The muscle tissues from 5 frozen samples were dissected before melted, and immediately transferred into 1 ml of QIAzol (from RNeasy Plus Universal Kit) and homogenised by T10 basic ULTRA-TURRAX (IKA, German). Total RNA was extracted by RNeasy Plus Universal Kit (QIAGEN, UK) according to the manufacturer’s protocol. All samples must meet requirements of the 260/280 ratio between 1.8 and 2.1, and the 260/230 ratio between 2.0 and 2.4, tested by using Nanodrop spectrophotometer (Thermo Fisher Scientific, USA). RNA integrity and concentration were assessed by using the RNA Nano 6000 Assay Kit of the Agilent Bioanalyzer 2100 system (Agilent Technologies, CA, USA), and Qubit RNA Assay Kit in Qubit 2.0 Flurometer (Life Technologies, CA, USA), respectively. Only the samples with a RIN value higher than 6.8 were further used. Moreover, RNA degradation and contamination were also monitored on 1% agarose gels. Clear bands of 28 s, 18 s and 5 s rRNA were needed.

### Library preparation, Illumina sequencing, and assembly

Six of these experimental groups were chosen for further comparative transcriptome analysis. Their experimental conditions and treatment identifiers are provided in Table 1. A total of 1.5 μg RNA per sample was used for the RNA sample preparations. Sequencing libraries were generated by using NEBNext Ultra RNA Library Prep Kit for Illumina (NEB, USA), and index codes were added to attribute sequences to each sample. TruSeq PE Cluster Kit v3-cBot-HS (Illumina) was used for the clustering of the index-coded samples performed on a cBot Cluster Generation System. Then, the library preparations were sequenced on an Illumina Hiseq platform and paired-end reads were generated. Clean data were obtained by removing reads containing adapter, reads containing ploy-N and low quality reads from raw data. Transcriptome assembly was then accomplished based on the clean data by using Trinity^45^. At last, transcripts were hierarchical clustering by Corset^46^.

**Table 1:** Experimental conditions of six experimental groups used for comparative transcriptome analysis.

Group ID	Temperature (°C)	Pressure (MPa)
T20P0.1	20	0.1
T20P15	20	15
T15P0.1	15	0.1
T15P15	15	15
T10P0.1	10	0.1
T10P15	10	15

### Gene functional annotation

All unigenes were annotated in seven databases, including NCBI non-redundant protein sequences (Nr), NCBI non-redundant nucleotide sequences (Nt), a manually annotated and reviewed protein sequence database (Swiss Prot), euKaryotic Ortholog Groups (KOG), Protein family (Pfam), Kyoto Encyclopedia of Genes and Genomes (KEGG), and Gene Ontology (GO). The annotation results of all unigenes are supplied in Table S1.

### Transcriptome differential expression analysis and enrichment analysis

First, FPKM and read count of six experimental groups were calculated by using RSEM^47^. Then, FPKM were normalised by using TMM method. The read counts and normalised FPKM of unigenes are supplied in Table S2. Second, differential expression analysis of paired-sample test was implemented by using DESeq. 2 package^48^ to identify differential expression genes (DEGs) involved in HHP acclimatisation. The following combinations were paired: T20P15 with T20P0.1, T15P15 with T15P0.1, and T10P15 with T10P0.1. Only genes with an adjusted *p*-value < 0.01 and |log2 (fold change)| > 1 were regarded as DEGs. In this study, only up-regulated genes were considered as activated genes in response to HHP exposure because only essential processes can be maintained, whereas nonessential processes are reduced outside the optimal range^49–53^.

The well-annotated DEGs were further analysed and they were grouped in 10 biological processes. The distance among experimental groups was calculated according to normalised FPKM of these well-annotated DEGs with vegan R package^54^ by using the euclidean method. Then, hierarchical clustering result was visualised by using pheatmap R package^55^ via the complete method. Moreover, the KEGG enrichment analysis of these up-regulated DEGs was implemented by using the KOBAS software^56^.

### Gene expression analysis and validation by quantitative real-time reverse transcription-PCR (qPCR)

A total of 23 DEGs were employed for qPCR by StepOnePlus Real-Time PCR System (Applied Biosystems, USA) to validate the RNA-seq results. Each 25 μl reaction contained 12.5 μl of FastStart Universal SYBR Green Master (Rox) (Roche, Switzerland), and 2.5 μl of template cDNA. The primer sequences (Table S3) were designed by Primer Premier 5.0 software (Premier Biosoft International, Palo Alto, CA, USA). DNase treatment was not required because gDNA solution (from RNeasy Plus Universal Kit) was used for RNA extraction. The cDNA library was established by PrimeScript II 1st Strand cDNA Synthesis Kit (Takara, Janpan) according to the manufacturer’s standard protocol.

Relative standard curve method was used for expression level analysis with *Rpl8* as internal control. The internal control was selected by GeNorm software (Primer Design, Ltd., Southampton University, Highfield Campus, Southampton Haunts, UK). Six 3-fold serial dilutions were performed on a cDNA template to ensure that each primer-set had a qPCR reaction efficiency of between 90 and 105% and a linearity greater than r^2^ = 0.98 across the predicted experimental cDNA concentration range. Normalised relative quantities were scaled to the control treatment (20 °C 0.1 MPa) in each comparison and converted to relative fold change (RFC). Then, log_2_ (RFC) was used to evaluate differential expression level. The melting curve analysis was performed at the end of each PCR to confirm only one PCR product was amplified. At last, Pearson correlation coefficients (PCC) between RNA-seq and qPCR results were calculated by corrplot R package^57^.

According to the results of differential expression analysis in this study, five of these genes, including glutamine synthetase (*GS*), phosphoenolpyruvate carboxykinase (*PEPCK*), peroxidase, crustin Pm5, and lysozyme, were selected and their expression patterns were examined in all experimental conditions by using qPCR. We also examined the expression level of a HHP induced gene *hsp70* (not existed in the result of transcriptome differential expression analysis in this study) which has been reported in many studies. Two-way ANOVA was used to determine whether temperature and hydrostatic pressure significantly impacted the gene expression patterns, and one-way ANOVA was used to determine whether hydrostatic pressure significantly impacted the gene expression patterns at each temperature condition. The ANOVA analyses were implemented by using R software^58^. The expression patterns of the six genes were shown on line graph by using Graphpad Prism 7 (Graphpad software, Inc., USA).

## Results

### Pressure tolerance of *Eogammarus possjeticus*

The highest examined hydrostatic pressure condition in the present study was 30 MPa. All experimental individuals died at any temperature condition after 16 h pressure exposure under 30 MPa (Table 2). The critical pressure of *E. possjeticus* is 25 MPa at temperature conditions from 10 °C to 20 °C because up to 50% individuals died under these conditions. However, 25 MPa in the low temperature of 5 °C resulted in 100% mortality rate. A total of 100% survival rate was observed at all temperature conditions (5–20 °C) after 16 h pressure exposure under 20 MPa.

**Table 2:** Mortality rates of *Eogammarus possjeticus* at different hydrostatic pressure and temperature conditions.

	0.1 MPa	5 MPa	10 MPa	15 MPa	20 MPa	25 MPa	30 MPa
20 °C	0%	0%	0%	0%	0%	50%	100%
15 °C	0%	0%	0%	0%	0%	40%	100%
10 °C	0%	0%	0%	0%	0%	40%	100%
5 °C	0%	0%	0%	0%	0%	100%	100%

Ten individuals were used in each experiment.

### Transcriptome sequencing, assembly and annotation

Six experimental groups were chosen for transcriptome analysis (Table 1). Qualities of sequencing are listed in Table S4. Clean reads were finally assembled into 138, 304 unigenes with a total length of 167, 172, 351 bp and an N50 length of 1, 900 bp (Table S5). A total of 60, 928 (44.05%) unigenes were annotated in at least one database (Table S6). Sequence analysis indicated that the experimental samples in the present study share 99% 16S rRNA sequence similarity with *Eogammarus possjeticus* according to Nt database.

### DEGs involved in acclimatisation of high pressure exposure

**Figure 1:**
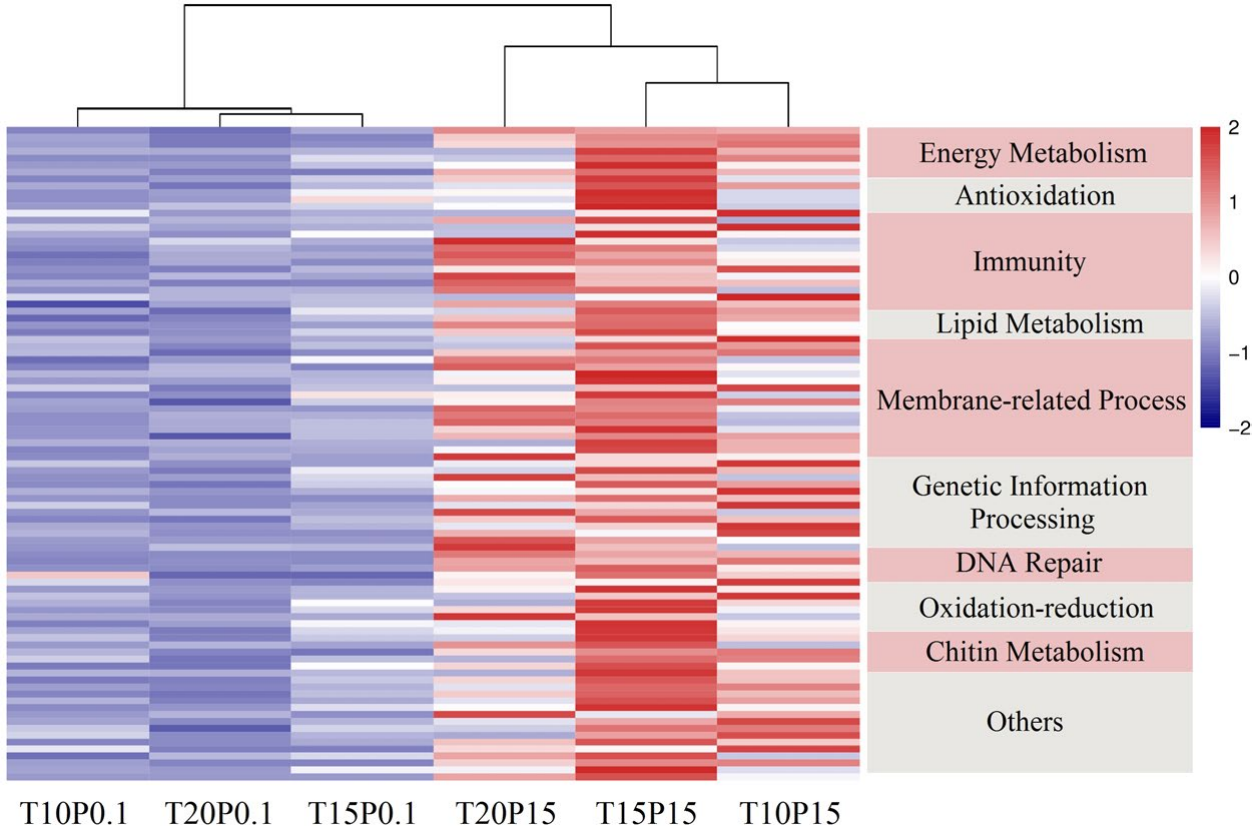
Expression patterns of 94 well-annotated differential expression genes. Heatmap Clustering is based on normalised FPKM of differential expression genes. Name of corresponded biological processes are listed on the right of the heatmap. T20P0.1: 20 °C, 0.1 MPa; T20P15: 20 °C, 15 MPa; T15P0.1: 15 °C, 0.1 MPa; T15P15: 15 °C, 15 MPa; T10P0.1: 10 °C, 0.1 MPa; T10P15: 10 °C, 15 MPa.

A total of 137 up-regulated genes were selected through DESeq. 2 paired test, of which 94 were well-annotated (Table S7). These genes were grouped into 10 different biological processes according to their functions as follows: energy metabolism (9 genes), antioxidation (5 genes), immunity (13 genes), lipid metabolism (4 genes), membrane-related process (18 genes), genetic information processing (13 genes), DNA repair (4 genes), oxidation-reduction (7 genes), chitin metabolism (6 genes) and others (15 genes). Their expression patterns were visualised via heatmap (Fig. 1). Results of KEGG enrichment indicated that six KEGG pathways were significant enriched (adjusted *p*-value < 0.01), including nitrogen metabolism, glutamatergic synapse, arginine biosynthesis, glyoxylate and dicarboxylate metabolism, GABAergic synapse,and alanine, aspartate and glutamate metabolism (Fig. S1).

Nine genes were grouped in energy metabolism. Four of them were annotated in glutamine synthetase (*GS*) which catalyse the amidation of glutamate to glutamine^59^. Two of them were annotated in phosphoenolpyruvate carboxykinase (*PEPCK*) which is a key enzyme in the lyase family involved in gluconeogenesis^60^. One gene was annotated in carbonic anhydrase (*CA*) which catalyse the conversion of CO_2_ to the bicarbonate ion and protons^61^.

Five genes were grouped in antioxidation. Three of them were annotated in vitellogenin (*VG*) which is known as a yolk protein and was reported to act as an antioxidant to promote longevity^62^. The other two genes were annotated in peroxidase and catalase respectively. Both genes catalyse the decomposition of hydrogen peroxide to water and oxygen^63,64^.

A total of 13 genes, including crustin Pm5, lysozyme, and alpha-2-macroglobulin (*α*_2_*M*), were grouped in immunity. The gene crustin Pm5 and lysozyme exhibit antimicrobial activity against some gram-positive bacteria^65,66^, and *α*_2_*M* serves as humoral defense barriers against pathogens^67^.

Four genes were grouped in lipid metabolism. They were fatty acid desaturase, elongation of very long chain fatty acids protein (*ELOVL*), sphingomyelin phosphodiesterase (*SMase*), and fatty acid-binding protein. Fatty acid desaturase is an enzyme which creates carbon-carbon double bond. It allows the membrane to become more fluid when the temperature is decreased^68^. A kind of *ELOVL* is required for the synthesis of C28 and C30 saturated fatty acids and of C28-C38 very long chain polyunsaturated fatty acids^69^. *SMase* is involved in sphingolipid metabolism^70^, and its activation is suggested to be involved in the production of ceramide in response to cellular stresses^71^. A total of 18 genes were grouped in membrane-related process, most of which are related to ion transmembrane transportation.

A total of 13 genes were grouped in genetic information processing, including DNA replication, transcription, and translation. Four genes were grouped in DNA repair and they were annotated in C_2_H_2_ zinc finger domain, MYM-type zinc finger domain, and C_4_ zinc finger domain. The functions of zinc finger proteins include DNA recognition, RNA packaging, and transcriptional activation^72^. Studies found that genes encoding for zinc finger domains expanded in deep-sea amphipod compared with other shallow-water species genes^15^.

### Gene expression analysis by qPCR

**Figure 2:**
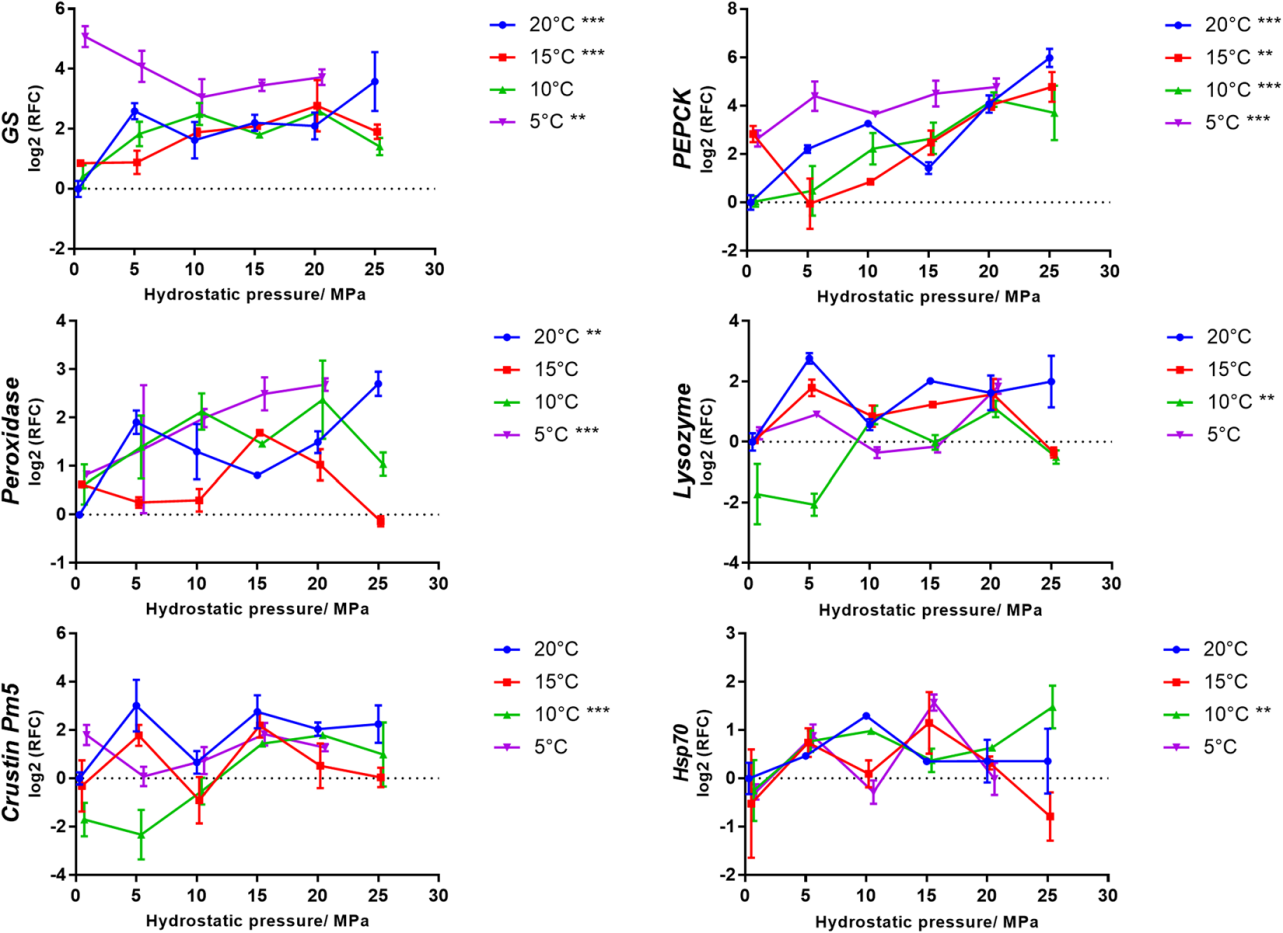
Effect of hydrostatic pressure on expression patterns of six genes at different temperature conditions. Normalised relative quantities were scaled to the control treatment (20 °C, 0.1 MPa) in each comparison and converted to relative fold change (RFC). Error bars represent 95% confidence intervals. Correlations between gene expression patterns and hydrostatic pressure at each temperature condition are tested by one-way ANOVA (***p-value < 0.001; **p-value < 0.01; *p-value < 0.05).

PCC between RNA-seq and qPCR results ranged from 0.77 to 0.99 (Table S3), which validated the reliability of the RNA-seq results. Two-way ANOVA results (Table 3) confirmed that the expression patterns of the five genes involved in pressure acclimatisation were significantly correlated with hydrostatic pressure, whereas *hsp70* was not. The expression patterns of four genes, including *GS*, *PEPCK*, peroxidase, and lysozyme, were also significantly affected by temperature. Pressure and temperature had a significant interaction on the expression patterns of two genes (*GS* and *PEPCK*). One-way ANOVA analysis (Table 3) indicated that although the gene expression level was not always significantly up-regulated by hydrostatic pressure, a positive correlation can be mostly observed between gene expression patterns and hydrostatic pressure (Fig. 2). Genes involved in energy metabolism (*GS* and *PEPCK*) can also be up-regulated by low temperature, whereas genes involved in immunity (crustin Pm5 and lysozyme) did not show this trend (Fig. 2).

**Table 3:** Correlations between gene expression patterns and two environmental variables tested by two-way ANOVA, and correlations between gene expression patterns and hydrostatic pressure at each temperature condition tested by one-way ANOVA.

	*GS*	*PEPCK*	Peroxidase	Crustin Pm5	Lysozyme	*Hsp70*
**Two-way ANOVA**						
Pressure	0.017^*^	2.44 × 10^−12***^	0.001^**^	0.001^**^	0.013^*^	0.115
Temperature	4.31 × 10^−7***^	0.003^**^	0.003^**^	0.083	5.58 × 10^−4***^	0.606
interaction	1.81 × ^10−5***^	0.034^*^	0.797	0.964	0.858	0.112
**One-way ANOVA**						
Pressure (20 °C)	2.96 × 10^−4***^	5.93 × 10^−6***^	0.002^**^	0.059	0.089	0.814
Pressure (15 °C)	1.17 × 10^−4***^	0.002^**^	0.976	0.857	0.567	0.663
Pressure (10 °C)	0.072	8.48 × 10^−9***^	0.167	7.39 × 10^−6***^	0.009^**^	0.002^**^
Pressure (5 °C)	0.006^**^	4.87 × 10^−4***^	9.11 × 10^−8***^	0.592	0.184	0.399

***p-value < 0.001; **p-value < 0.01; *p-value < 0.05.

## Discussion

The utilisation of a pressure vessel provided a stable and controllable experimental context allowing for pressure tolerance to be examined accurately. Although 5 d are enough for insect and amphibian to acclimate to a temperature variation of 5 °C, a longer period of acclimatisation time of more than 10 d is recommended in marine invertebrates^73^. We measured water quality before and after each experiment to ascertain that the experimental context only involved pressure and temperature stresses, and no significant difference was observed. Therefore, hydrostatic pressure and temperature were the only two variables.

### Pressure tolerance of *Eogammarus possjeticus*

The shallow-water amphipod *E. possjeticus* can survive 100% at 20 MPa at all temperature conditions ranging from 5 °C to 20 °C for at least 16 h. The impressive tolerance of HHP was also observed among many other shallow-water invertebrates (for review see Brown & Thatje 2014^6^). Remarkably, the pressure boundary of most studied shallow-water invertebrates ranged from 20 MPa to 25 MPa, coinciding with regions of high species turnover^5,6^.

Low temperatures lead to a decreasing pressure tolerance of *E. possjeticus*. The phenomenon of decreasing temperature resulting in a reduction of pressure tolerance was also reported among other marine animals. For example, the amphipod *Stephonyx biscayensis*, subjected to a 10-minute pressure exposure, can tolerate 30 MPa at 10 °C; however, its pressure tolerance fell to 20 MPa at 3 °C^74^. Increasing the pressure by 1 MPa every 5 min up to 30 MPa resulted in the rate of “loss of equilibrium” in ≥50% of shrimp *Palaemonetes varians* observed at 20 MPa at 20 °C and 15 MPa at 10 °C^75^.

### Genes in response to high hydrostatic pressure

Most deep-sea animals are widely accepted to have originated from shallow waters as a consequence of a series of extinction events during the Phanerozoic, and the colonisation of deep sea by shallow-water organisms that fallowed^76,77^. Most existing studies focused on these multiple colonisation events, and the adaptation mechanisms of deep-sea species. However, the deep-sea environments acclimatisation mechanisms of shallow-water fauna are seldom studied. This question is of great importance in the present context of climate change and ocean warming, which are likely to force bathymetric migrations of marine fauna^8,11^.

In the present study, we explored the acclimatisation mechanisms of the shallow-water amphipod *E. possjeticus* via transcriptome analysis for the first time. Our results suggested that several biological processes, including energy metabolism, antioxidation, immunity, lipid metabolism, membrane-related process, genetic information processing, and DNA repair, are involved in the acclimatisation of HHP.

The HHP condition of 15 MPa is apparently beyond optimum for the shallow-water amphipod *E. possjeticus*. Homeostatic effort is required to maintain internal conditions within their physiological tolerance boundaries. Consequently, an increased level of energy requirement to facilitate the increased homeostatic effort is needed. Our results identified three genes are involved in energy metabolism, and two of them (*PEPCK* and *GS*) involved in energy production. *PEPCK*, involved in pathway of gluconeogenesis^60^, converts oxaloacetate into phosphoenolpyruvate, which has the highest energy phosphate bond found in living organisms. *GS* catalyse the amidation of glutamate to glutamine^59^ which is the most abundant amino acid in the plasma and plays an essential role in protein and lipid synthesis^78,79^. Moreover, glutamine acts as an important energy source in cells^80–82^, and glutaminolysis partially recruits reaction steps from the citric acid cycle. An increased oxygen demand is required to support a high level of energy requirement. Oxygen is converted into carbon dioxide through aerobic respiration, resulting in the up-regulation of *CA*, which converts CO_2_ to the bicarbonate ion and protons^61^.

Although the metabolic rate of *E. possjeticus* was not examined in this study, existing studies on crab *Lithodes maja* indicated that oxygen consumption increases with increasing hydrostatic pressure and is significantly higher at 7.5–17.5 MPa than at 0.1 MPa^11^. The Pressure tolerance is constrained by oxygen concentration^8,11^. It may be because the oxygen intake of animals has its ceiling. However, the oxygen consumption of deep-sea species did not appear to be elevated compared with shallow-water congeneric species^83–85^, suggesting that the former are functionally adapted to high hydrostatic pressure and low temperature^86^. Although the oxygen consumption of deep-sea species is not significantly higher than that of shallow-water species, the genes involved in carbohydrate and energy metabolism are positively selected in amphipod *Hirondellea gigas*, which lives at the depth of 10, 929 m in the Challenger Deep^15^. In general, energy metabolism is of great important in both acclimation and adaption to the deep sea.

Increased mitochondrial activity is needed to meet the energy requirement under high pressure stress. Therefore, mitochondrial oxidative damage is elevated. Consequently, genes involved in antioxidation may be activated. Moreover, the electron transport chain of many reactions would be impeded because the enzyme activity is highly impacted by high pressure^87^, which consequently induces antioxidant response. In this study, five genes involved in HHP acclimatisation, including peroxidase and catalase, are grouped in antioxidation. An increasing number of evidence indicates that antioxidant defense responses can be induced by HHP and low temperature^88–90^. Experimental evidence indicates that the mutant of bacteria *Shewanella piezotolerans*, with enhanced antioxidant defense capacity by experimental evolution under H_2_O_2_ stress, has better growth ability at the high pressure of 20 MPa and low temperature of 4 °C than the wild type *S. piezotolerans*^17^.

Elevated oxidative damage leads to apoptosis^91^. Thus, the immune system would be activated in response to mtDNA released from apoptotic mitochondria^92^. Moreover, misfolded proteins that have resulted from high pressure also induce an immune response^93^. In the present study, a total of 13 up-regulated DEGs were grouped in immunity, including crustin Pm5 and lysozyme, both of which have antimicrobial activities and serve as part of the innate immune system^65,66^. Genes involved in immunity were positive selected in the deep-sea urchin *Allocentrotus fragilis* genome compared with shallow-water urchin *Strongylocentrotus purpuratus*^19^, and was up-regulated in the deep-sea shrimp *Rimicaris sp*. compared with the same species maintained at atmospheric pressure for 10 d^94^. However, *hsp70* was not significantly induced by hydrostatic pressure exposure in the present study. Heat shock proteins play essential roles in heat shock tolerance and the refolding of denatured proteins. They also respond to a variety of stressors, such as pathogen infection, oxidative stress, heavy metals, and other xenobiotics^95^. A study on the gene *hsp70* of shrimp *Palaemonetes varians* found that the expression level measured in 1 h was three times higher than that observed in shrimp maintained for 7 d under similar temperature and high pressure conditions^10^. Therefore, the expression level of *hsp70* is related to time period under hydrostatic pressure exposure, and its up-regulation is probably a universal stress response in a short time period.

High pressure leads to a reduction of membrane fluidity^21–25^. The brain gangliosides of the deep-living fish are rich in mono-unsaturated fatty acids and low in saturated fatty acids^96^. The homeoviscous adaptation of membranes is an important component of adaptation to depth^97^. Moreover, studies on homeoviscous acclimation to pressure on microorganisms found that the increasing proportion of unsaturated fatty acid and branched-chain fatty acid can remit the reduction of membrane fluidity imposed by high pressure and low temperature^27,31^. In our studies, four genes were grouped in lipid metabolism, and two of them are involved in the synthesis of unsaturated fatty acids, including *fatty acid desaturase*^68^ and *ELOVL*^69^. Thus, homeoviscous acclimatisation is important for the deep sea acclimation of shallow-water species.

Almost all membrane-based functions are affected by high pressures^98^, and transmembrane ion flux is extraordinarily sensitive to pressure. The tansmembrane protein Na^+^-K^+^-adenosine triphosphatase (*Na*^+^*-K*^+^*-ATPase*), which plays a key role in osmoregulation, varies its activity in accordance with the fluidity of the surrounding membrane lipids^99^. Studies about *Na*^+^*-K*^+^*-ATPase* of teleost gills found that the enzyme from the deep-living fishes is far less inhibited by pressure than the homologous enzymes of shallow-living species. The evolution of *Na*^+^*-K*^+^*-ATPase* of deep-sea fish might be a consequence of homeoviscous adaptation of membranes. In our results, thirteen genes belong to ion transmembrane transportation genes, five of which involved in Na^+^ transmembrane transportation. This tendency reflects that ion transportation in *E. possjeticus* was impeded under high pressure, and the maintenance of its normal function is of great importance for pressure acclimatisation.

## Conclusions

This study reveals that the shallow-water amphipod *E. possjeticus* can survive 100% under 20 MPa for at least 16 h at temperature conditions from 5 °C to 20 °C. Decreasing temperature results in a reduction of pressure tolerance of *E. possjeticus*. We identified several genes and biological processes involved in the acclimatisation of shallow-water invertebrates in deep-sea environments, including energy metabolism, antioxidation, immunity, lipid metabolism, membrane-related process, genetic information processing, and DNA repair.

### Ethical approval and informed consent

This study did not involve any endangered or protected species and followed all relevant ethical guideline. The samples examined in this study were used as aquacultural feed in China.

## Supplementary information

Figure S1

Table S1

Table S2

Table S3

Table S4

Table S5

Table S6

Table S7

## Data Availability

Relevant data supporting this manuscript are contained within the tables of this manuscript or provided in the supplementary material. The clean data of RNA-seq were available from National Center for Biotechnology Information Sequence Read Archive database (SRA accession numbers: SRR7205161, SRR7205163, SRR7205164, SRR7205158, SRR7205159, SRR7205160).The unigenes were submitted to the Transcriptome Sequencing Assembly database (TSA accession number: SUB4070551).
