# Supplementary material for: Comparative transcriptome analysis of Eogammarus possjeticus at different hydrostatic pressure and temperature exposures
Source: Sci Rep. 2019 Mar 5;9:3456. doi: 10.1038/s41598-019-39716-y (PMC6401005; doi:10.1038/s41598-019-39716-y)
Supplement: Supplementary file 1 — Figure S1 [file 41598_2019_39716_MOESM1_ESM.pdf]

Title page

Comparative transcriptome analysis of *Eogammarus possjeticus* at different hydrostatic pressure and temperature exposures

Jiawei Chen[1, 2], Helu Liu[1], Shanya Cai[1, 2] and Haibin Zhang[1, *]

[1] Institute of Deep-sea Science and Engineering, Chinese Academy of Sciences, Sanya 572000, China

[2] University of Chinese Academy of Sciences, Beijing 100049, China

***Author for correspondence:** Haibin Zhang, Institute of Deep-sea Science and Engineering, Chinese Academy of Sciences, Sanya 572000, China

E-mail: hzhang@idsse.ac.cn

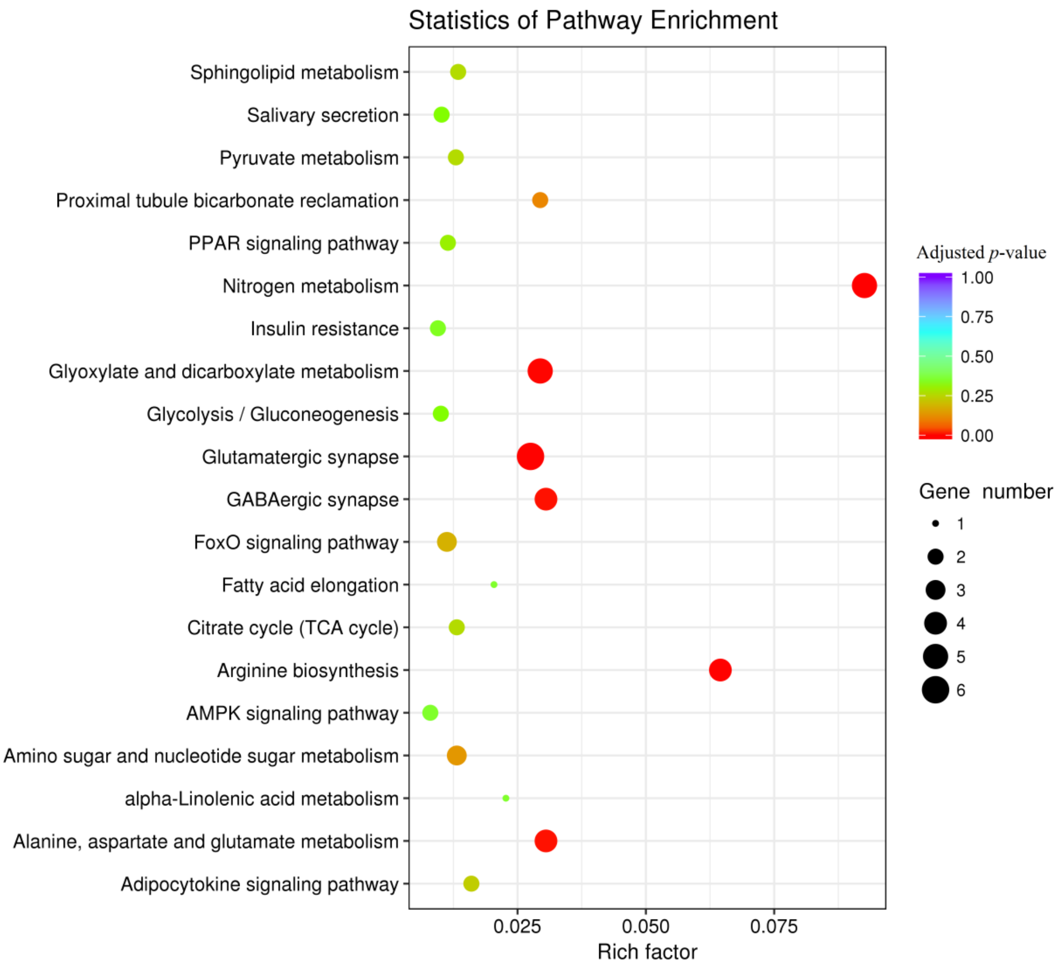

Figure S1. Results of KEGG enrichment of 94 well-annotated differential expression genes. KEEG: Kyoto Encyclopedia of Genes and Genomes.
