# Supplementary material for: Comparative transcriptome analysis of Eogammarus possjeticus at different hydrostatic pressure and temperature exposures
Source: Sci Rep. 2019 Mar 5;9:3456. doi: 10.1038/s41598-019-39716-y (PMC6401005; doi:10.1038/s41598-019-39716-y)
Supplement: Supplementary file 4 — Table S3 [file 41598_2019_39716_MOESM4_ESM.pdf]

Title page

Comparative transcriptome analysis of *Eogammarus possjeticus* at different hydrostatic pressure and temperature exposures

Jiawei Chen[1, 2], Helu Liu[1], Shanya Cai[1, 2] and Haibin Zhang[1, *]

[1] Institute of Deep-sea Science and Engineering, Chinese Academy of Sciences, Sanya 572000, China
[2] University of Chinese Academy of Sciences, Beijing 100049, China

***Author for correspondence:** Haibin Zhang, Institute of Deep-sea Science and Engineering, Chinese Academy of Sciences, Sanya 572000, China
E-mail: hzhang@idsse.ac.cn

Table S3. Information of the primers used in quantitative real-time reverse transcription-PCR (qPCR) analysis (with *Rpl8* as internal control). PCC: Pearson correlation coefficients between qPCR and RNA-Seq.

Gene name	Gene ID	PCC	5'- 3' forward primer	5'- 3' reverse primer
Rpl8	Cluster-13637.43027		GCAGCAAGAAGATCCTCCCGTCCAA	ACACGGCAGGCAAACGAATCCAACG
GS	Cluster-13637.55967	0.90	GCCATCCACATATCGTCACCCAT	CACTCGCCAAAGACCAACATCCT
PEPCK	Cluster-13637.35569	0.86	ATTGTCTTTCGGGTCAGGCTACGGT	AGTGAAGGAGTCATCATCGCCAGGT
Peroxidase	Cluster-13637.45510	0.99	AGGTGCCGTCTATGGTGCGGTATTT	CCACGAACAGGCTCTTCAGGTCAA
Crustin pm5	Cluster-13637.44495	0.96	ACGATGGCAGGCTTGCATGAGTG	TGTTGCTGGCGTGTGTAGTGGCT
Lysozyme	Cluster-13637.42497	0.90	TTACAACATTACCACCAACAAGG	ACCAGTCGGACAGAAACAGAGGCTA
Hsp70	Cluster-13637.44177	0.93	TCCACTGCTGGTGACACTCATCTT	ATCGGCACAGAGCTCCTCAAACCTA
CA	Cluster-13637.39561	0.98	GCTGCCCTGGTAGCGGTAGAAAT	TTGGATGACGCCCTGGGCCACGAAGAC
A2M	Cluster-13637.43635	0.89	GTAACTGACGATCCCCTGTTGAAAGA	AGAAACCAGAAGCACCCAAACCACT
ATP1A	Cluster-13637.41266	0.84	CATCGCTAAGGAGATCGCTCATTTC	ACATGTGGGCGACAGTCATACGGTT
CALM	Cluster-13637.38529	0.82	TCCACCTCCTCGTCTGTCAACTTCTC	GACGGTAACGGCACAATCGACTTCC
Peritrophin	Cluster-13637.45283	0.85	GAGCAACTACGAGTGTCCCAAGCCA	CCATCTCCTCCGCATTTCTCAGTGT
Clottable protein	Cluster-13637.43258	0.90	AGTTAGTCCGAATCCGCTCACAATC	CCTCACCAACATCACTTCTACCTCCTT
IDH3	Cluster-13637.43931	0.88	TTGTGCACGGCGGTGACTTTCTTAC	GCAATGGAATCTGCCTGAAGGGAAT
Enolase	Cluster-13637.42843	0.77	CTGCTCACTTCATTCCTTCCACACC	GCTCAGAGAGGCTGGCCAAGTACAA
HmgA	Cluster-13637.51258	0.90	GATGGGTTTTGTCACCTCTGTCTT	TGTCCATTTGGGAGTAGAAGCGA
Ring finger	Cluster-13637.39981	0.80	TTCAATCGAGGACGGAGAGATGGAC	CGAGTACGACAGCACGAGCACAC
Vitellogenin	Cluster-13637.41706	0.97	AACTACTCAAGCAGAACCGAACATT	TCTTCAGTATCGGCTCCCTCAGTTG
WAP	Cluster-13637.37987	0.89	GCCTTCTGTCCTCATGCAGGTGTAG	CTCGCTGTGCTGCTGGTCGTGT
MDH	Cluster-13637.45447	0.79	GCACCCTCCATCCCCAAGGAAAACT	TTCTTCACCTCGGAGACAGGCACCC
ATPeV0A	Cluster-13637.35182	0.99	CAACAAGTTCACCTTCGGCTTCCAG	ACGCTCCTTCAGCACCATCCACAGA
ATPeV1B	Cluster-13637.60972	0.96	ATGAAGGCTGTGGTGGGCGAAGAAG	CACACAAACCCAATAAACCTACGAG
ATPeV1C	Cluster-13637.45517	0.99	CCTTGCTGAAGAGGGTGACCGAGAA	GGGAGACCTGGTGAAGAAAAGTGAC
AMP	Cluster-13637.45969	0.92	TTGGGTGAGTAAGGATTCGCATTGG	ACGCATACTTGGTAATGACGACTAA
