# Supplementary material for: Comparative transcriptome analysis of Eogammarus possjeticus at different hydrostatic pressure and temperature exposures
Source: Sci Rep. 2019 Mar 5;9:3456. doi: 10.1038/s41598-019-39716-y (PMC6401005; doi:10.1038/s41598-019-39716-y)
Supplement: Supplementary file 5 — Table S4 [file 41598_2019_39716_MOESM5_ESM.pdf]

Title page

Comparative transcriptome analysis of *Eogammarus possjeticus* at different hydrostatic pressure and temperature exposures

Jiawei Chen[1, 2], Helu Liu[1], Shanya Cai[1, 2] and Haibin Zhang[1, *]

[1] Institute of Deep-sea Science and Engineering, Chinese Academy of Sciences, Sanya 572000, China
[2] University of Chinese Academy of Sciences, Beijing 100049, China

***Author for correspondence:** Haibin Zhang, Institute of Deep-sea Science and Engineering, Chinese Academy of Sciences, Sanya 572000, China
E-mail: hzhang@idsse.ac.cn

Table S4. Quality of sequencing. T20P0.1: 20 ℃, 0.1 MPa; T20P15: 20 ℃, 15 MPa; T15P0.1: 15 ℃, 0.1 MPa; T15P15: 15 ℃, 15 MPa; T10P0.1: 10 ℃, 0.1 MPa; T10P15: 10 ℃, 15 MPa.

Sample	Raw Reads	Clean reads	Clean bases	Error(%)	Q20(%)	Q30(%)	GC(%)
T20P0.1	103,330,674	98,730,068	14.81G	0.02	95.41	88.83	42.93
T20P15	108,885,900	104,024,668	15.6G	0.02	95.57	89.16	42.15
T15P0.1	104,172,774	98,208,320	14.73G	0.02	95.35	88.76	43.1
T15P15	97,029,846	92,706,622	13.91G	0.03	95.24	88.52	43.01
T10P0.1	98,752,258	94,307,414	14.15G	0.02	95.26	88.48	40.23
T10P15	99,222,460	93,812,168	14.07G	0.02	95.99	90.27	42.57
