# Supplementary material for: Comparative transcriptome analysis of Eogammarus possjeticus at different hydrostatic pressure and temperature exposures
Source: Sci Rep. 2019 Mar 5;9:3456. doi: 10.1038/s41598-019-39716-y (PMC6401005; doi:10.1038/s41598-019-39716-y)
Supplement: Supplementary file 6 — Table S5 [file 41598_2019_39716_MOESM6_ESM.pdf]

Title page

Comparative transcriptome analysis of *Eogammarus possjeticus* at different hydrostatic pressure and temperature exposures

Jiawei Chen[1, 2], Helu Liu[1], Shanya Cai[1, 2] and Haibin Zhang[1, *]

[1] Institute of Deep-sea Science and Engineering, Chinese Academy of Sciences, Sanya 572000, China

[2] University of Chinese Academy of Sciences, Beijing 100049, China

***Author for correspondence:** Haibin Zhang, Institute of Deep-sea Science and Engineering, Chinese Academy of Sciences, Sanya 572000, China

E-mail: hzhang@idsse.ac.cn

Table S5. Quality of assembly results, unit: bp.

	Total number	Min Length	Mean Length	Median Length	Max Length	N50	N90	Total Nucleotides
Transcripts	277, 240	201	743	347	18, 279	1, 422	264	205, 886, 880
Unigenes	138, 304	201	1, 202	739	18, 279	1, 900	518	167, 172, 351
