# Supplementary material for: Comparative transcriptome analysis of Eogammarus possjeticus at different hydrostatic pressure and temperature exposures
Source: Sci Rep. 2019 Mar 5;9:3456. doi: 10.1038/s41598-019-39716-y (PMC6401005; doi:10.1038/s41598-019-39716-y)
Supplement: Supplementary file 7 — Table S6 [file 41598_2019_39716_MOESM7_ESM.pdf]

Title page

Comparative transcriptome analysis of *Eogammarus possjeticus* at different hydrostatic pressure and temperature exposures

Jiawei Chen[1, 2], Helu Liu[1], Shanya Cai[1, 2] and Haibin Zhang[1, *]

[1] Institute of Deep-sea Science and Engineering, Chinese Academy of Sciences, Sanya 572000, China
[2] University of Chinese Academy of Sciences, Beijing 100049, China

***Author for correspondence:** Haibin Zhang, Institute of Deep-sea Science and Engineering, Chinese Academy of Sciences, Sanya 572000, China
E-mail: hzhang@idsse.ac.cn

Table S6. Summary of annotation results of unigenes in seven databases, including NCBI non-redundant protein sequences (Nr), NCBI non-redundant nucleotide sequences (Nt), A manually annotated and reviewed protein sequence database (Swiss Prot), euKaryotic Ortholog Groups (KOG), Protein family (Pfam), Kyoto Encyclopedia of Genes and Genomes (KEGG), and Gene Ontology (GO).

	Number of unigenes	Percentage (%)
Annotated in Nr	42, 726	30.89
Annotated in Nt	11, 510	8.32
Annotated in KEGG	21, 632	15.64
Annotated in Swiss Prot	34, 333	24.82
Annotated in Pfam	47, 686	34.48
Annotated in GO	48, 339	34.95
Annotated in KOG	21, 552	15.58
Annotated in all Databases	6, 090	4.40
Annotated in at least one Database	60, 928	44.05
